# Early versus Deferred Treatment for Smoldering Multiple Myeloma: A Meta-Analysis of Randomized, Controlled Trials

**DOI:** 10.1371/journal.pone.0109758

**Published:** 2014-10-03

**Authors:** Minjie Gao, Guang Yang, Van S. Tompkins, Lu Gao, Xiaosong Wu, Yi Tao, Xiaojing Hu, Jun Hou, Ying Han, Hongwei Xu, Fenghuang Zhan, Jumei Shi

**Affiliations:** 1 Department of Hematology, Shanghai Tenth People’s Hospital, Tongji University School of Medicine, Shanghai, China; 2 Department of Pathology, University of Iowa Carver College of Medicine, Iowa City, Iowa, United States of America; 3 Department of Internal Medicine, University of Iowa Carver College of Medicine, Iowa City, Iowa, United States of America; University of Kentucky, United States of America

## Abstract

**Purpose:**

Whether patients with smoldering multiple myeloma (SMM) needed to receive early interventional treatment remains controversial**.** Herein, we conducted a meta-analysis comparing the efficacy and safety of early treatment over deferred treatment for patients with SMM.

**Methods:**

MEDLINE and Cochrane Library were searched to May 2014 for randomized controlled trials (RCTs) that assessed the effect of early treatment over deferred treatment. Primary outcome measure was mortality, and secondary outcome measures were progression, response rate, and adverse events.

**Results:**

Overall, 5 trials including 449 patients were identified. There was a markedly reduced risk of disease progression with early treatment (Odds Ratio [OR] = 0.13, 95% confidence interval [CI] = 0.07 to 0.24). There were no significant differences in mortality and response rate (OR = 0.85, 95% CI = 0.45 to 1.60, and OR = 0.63, 95% CI = 0.32 to 1.23, respectively). More patients in the early treatment arm experienced gastrointestinal toxicities (OR = 10.02, 95%CI = 4.32 to 23.23), constipation (OR = 8.58, 95%CI = 3.20 to 23.00) and fatigue or asthenia (OR = 2.72, 95%CI = 1.30 to 5.67). No significant differences were seen with the development of acute leukemia (OR = 2.80, 95%CI = 0.42 to 18.81), hematologic cancer (OR = 2.07, 95%CI = 0.43 to 10.01), second primary tumors (OR = 3.45, 95%CI = 0.81 to 14.68), nor vertebral compression (OR = 0.18, 95%CI = 0.02 to 1.59).

**Conclusions:**

Early treatment delayed disease progression but increased the risk of gastrointestinal toxicities, constipation and fatigue or asthenia. The differences on vertebral compression, acute leukemia, hematological cancer and second primary tumors were not statistically significant. Based on the current evidence, early treatment didn’t significantly affect mortality and response rate. However, further much larger trials were needed to provide more evidence.

## Introduction

Multiple myeloma (MM) is a cancer of plasma cells that accumulate in the bone marrow and produce a monoclonal protein [1]. It is the second most common hematologic cancer, accounting for 15–20% of deaths from hematologic cancer and about 2% of deaths from cancer [1], [2]. Advances in the basic understanding of MM and the introduction of novel agents including thalidomide, lenalidomide and bortezomib, have delayed progression and prolonged patients’ survival. Despite these advances, unfortunately there is still no cure for MM, and the goal of therapy is to prolong survival [3], [4]. Prognosis of patients with MM depends mainly on the stage of the disease [5].

According to the International Myeloma Working Group guidelines, MM-related neoplastic conditions have been categorized into monoclonal gammopathy of undetermined significance (MGUS), smoldering (asymptomatic) myeloma (SMM), solitary plasmacytoma of bone, and symptomatic multiple myeloma [6]. Diagnosed symptomatic multiple myeloma falls into one of three stages, IB, II, or IIIA/B, whereas SMM is identified as stage IA myeloma as determined by Durie/Salmon staging [6]. SMM patients are at high risk of progressing to symptomatic myeloma, with a median time to progression of approximately 1–2 years [7]. This intermediate condition is characterized by monoclonal protein concentrations of more than or equal to 3 g/dL and/or bone marrow plasma cells that are more than or equal to 10% without myeloma related organ or tissue impairment [8]–[10]. 54% of SMM patients will progress to frank multiple myeloma 5 years after initial diagnosis. This value exceeds 70% 15 years post-diagnosis [11], [12].

Historically, the majority of patients with SMM are followed without therapy until symptoms developed [13], [14]. The main reason for this conservative approach was that previous options for MM therapy were limited and toxic and unable to improve survival benefit [5], [15] based on the conclusions from three small, randomized controlled trials prior to 2003 [16]–[18]. These trials compared early treatment with melphalan and prednisone with observation and demonstrated a significant delay in time to progression. However, this did not translate into better overall survival. Because all the trials included in the He et al. meta-analysis [5] used non-targeted agents (melphalan and prednisone) and had small sample sizes, the treatment of SMM remained controversial [12]. A better understanding of the biology of MM has led to a wealth of novel agents to treat it, providing for the possibility that at least some of these new agents may be used to effectively intervene at early stages of MM. Investigators have since begun to carry out clinical trials to evaluate novel drugs and new combinations of existing treatments in SMM. From the year 2003 onwards, two phase randomized, controlled trials (RCTs) have been completed [19], [20] and one phase III RCT with 370 SMM patients is ongoing. Immuno-modulatory agents (IMiDs) were used in each of these three more recent RCTs.

To gain a better, more complete and current understanding of the efficacy and safety of early treatment over deferred treatment for SMM patients, we conducted a meta-analysis based on data from the five completed phase III RCTs. Our analysis includes the three trials [16]–[18] previously used by He et al. [5] but also include the two more recent trials that used IMiDs [19], [20]. In performing our meta-analysis we address whether early treatment of SMM with the newer, targeted agents (IMiDs) have improved clinical benefit over the non-targeted agents (melphalan and prednisone), and whether increasing the sample size provides more reliable data for early treatment regardless of agent type.

## Methods

### Search strategy

We used MEDLINE and Cochrane Library to locate all relevant studies published up to May 2014. The search criterion was listed in Table S1 and Table S2. We used ‘the related articles’ function in PubMed to identify other potentially relevant articles. Further, we searched the ClinicalTrials.gov registry. We also checked all the references of retrieved articles and asked for additional data and explanations when key information relevant to the meta-analysis was missing. The data was collected only from published, peer-reviewed papers.

### Selection criteria

Studies included in our analyses were required to be RCTs that compared early versus deferred treatment for SMM. The treatment strategy and the criteria used for selecting patients also needed to be reported. Further, every RCT must have either reported clinical outcomes or safety of the treatments. The eligibility of each study was assessed independently by two investigators.

### Data extraction and methodological quality assessment

The quality of trials was evaluated by two independent reviewers by examining the adequacy of the allocation randomization, allocation concealment, blinding, data analysis, withdrawals and dropouts and power analysis. Two reviewers performed data extraction independently based on selection criteria. If a disagreement arose, agreement was achieved through consultation with a third reviewer.

### Outcomes Assessments

Our primary outcome for this meta-analysis was mortality. Secondary outcomes included progression, response rate and adverse events. Mortality was defined as time from randomization to death from any cause. Progression was defined as time from randomization to documentation of progression. Safety outcomes included the incidence of adverse events, specifically gastrointestinal toxicities, constipation, fatigue or asthenia, vertebral compression, acute leukemia, hematological cancer and second primary tumor.

### Statistical analysis

The effect of treatment for each study was expressed as Odds Ratio [OR] of the early treatment arm over deferred treatment arm, with 95% confidence interval [CI]. A random-effect model was obtained to conduct a meta-analysis of all the relevant RCTs to get a conservative conclusion. Heterogeneity was assessed by both chi-squared test and I^2^ statistics. Statistically significant heterogeneity was defined as p-value <0.10 or I^2^ statistic>50%. Revman software (5.2) was used to perform all calculations.

## Results

### 1 Selection of the trials

**Figure 1:**
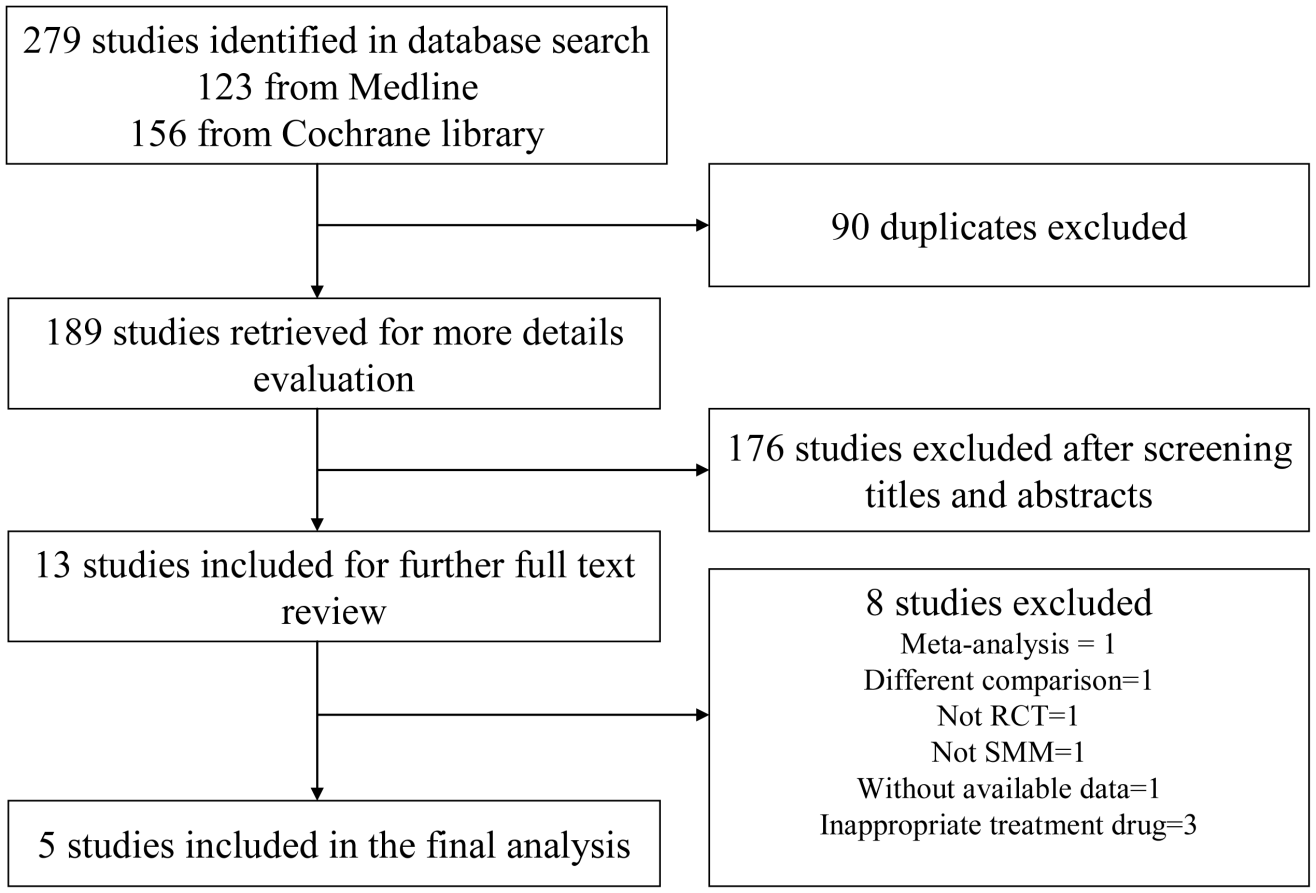
Flowchart of the selection of studies.

Our initial search yielded 279 potentially relevant studies, of which 90 studies were duplicated and 176 studies were deemed ineligible after screening titles and abstracts (Figure 1). The full text of the remaining 13 studies was reviewed in full, of which five randomized controlled trials fully met the inclusion criteria [16]–[20]. Excluded full-text studies, with the reasons for exclusion, were listed in Table S3.

### 2 Description of trials

An outline of the five trials is provided in Table 1. The trial results were published between 1993 and 2013. A total of 449 patients with SMM were included. Three of the trials used melphalan and prednisone as early treatment agents [16]–[18]. Two of the trials used IMiDs as early treatment agents [19]–[20]. The methodological quality of the 5 trials is summarized in Table 2. Two trials described the methods of randomization and allocation concealment [17], [18]. Three trials used an intention-to-treat analysis [16], [19], [20]. None of the five trials described power analysis and reported double blinding of the participants and outcome assessors. All trials described withdrawls and dropouts [16]–[20].

**Table 1:** Characteristics of studies fulfilling inclusion criteria in the meta-analysis.

Author[year]	Disease	Early treatmentdefined as	Deferred treatmentdefined as	No. of enrolled/analyzed patients	Intervention
Hjorth[1993]	SMM	Immediate treatment ondiagnosis/randomization	Observation untilsymptomatic diseaseprogression	E: 25/25 D: 25/25	M: 0.25 mg/kg P: 2 mg/kg d1-4 of 6 w intervals
Riccardi[1994]	SMM	Immediate treatment ondiagnosis/randomization	Observation untilsymptomatic diseaseprogression	E:38/34 D:40/40	M: 0.21 mg/kg d1-4 of 6 w intervals P:0.5 mg/kg d1-10 of 6 w intervals
Riccardi[2000]	SMM	Immediate treatment ondiagnosis/randomization	Observation untilsymptomatic diseaseprogression	E:75/72 D:70/66	M: 0.21 mg/kg d1-4 of 6 w intervals P:0.5 mg/kg d1-10 of 6 w intervals
Witzig[2013]	SMM	Immediate treatment ondiagnosis/randomization	Observation untilsymptomatic diseaseprogression	E:35/35 D:33/33	ZLD: 4 mg/d Thal: 200 mg/d a 28 d cycle
Mateos[2013]	High-riskSMM	Immediate treatment ondiagnosis/randomization	Observation untilsymptomatic diseaseprogression	E:57/57 D:62/62	Induction (L: 25 mg/d d1-21 Dex:20 mg/d d1-4,12–15 4 w intervals, 9 cycles)Maintenance (L: 10 mg/d d1-21, a 28 d cycle, 2 y)

MM: multiple myeloma; SMM: smouldering myeloma; M: melphalan; P: prednisone; ZLD: zoledronic acid; Thal: thalidomide; L: lenalidomide; Dex: dexamethasone; E: early treatment arm; D: deferred treatment arm; d: day; w: week; y: year.

**Table 2:** Methodological quality assessment of included trial.

Author [year]	Allocation generation	Allocationconcealment	Doubleblinding	ITT	Withdrawls anddropouts described	Power analysisdescribed
Hjorth [1993]	Unclear	Unclear	No	Yes	Yes	No
Riccardi [1994]	Computer generated	Adequate	No	No	Yes	No
Riccardi [2000]	Computer generated	Adequate	No	No	Yes	No
Witzig [2013]	Unclear	Unclear	No	Yes	Yes	No
Mateos [2013]	Unclear	Unclear	No	Yes	Yes	No

ITT: intention-to-treat.

### 3 Mortality

**Figure 2:**
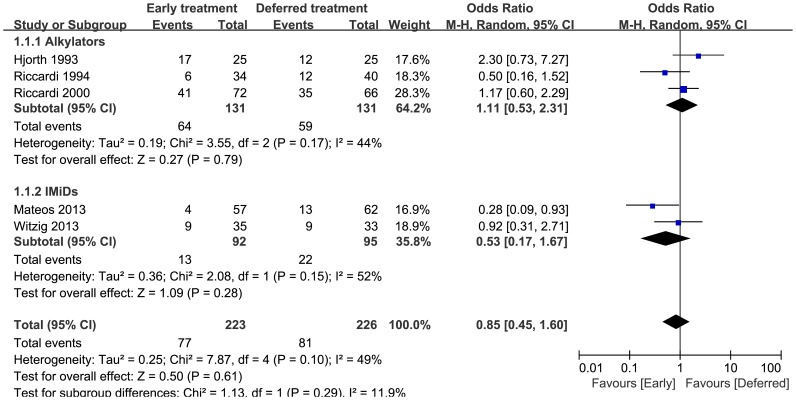
Mortality odds ratios of early treatment versus deferred treatment. The 95% CIs are shown. Squares represent the odds ratios and 95% CI for alkylators or IMiDs and finally for the overall estimate. CIs, confidence intervals; IMiDs, immuno-modulatory agents.

In the early treatment and deferred treatment arms, 223 and 226 patients were evaluable for mortality, respectively. In total, there were 77 deaths in the early treatment arm and 81 deaths in the deferred treatment arm. As shown in Figure 2, no statistically significant heterogeneity was observed (p = 0.10, I^2^ = 49%), and there was no significant difference in overall mortality between the two arms (pooled OR = 0.85, 95%CI = 0.45 to 1.60). When stratifying the data according to treatment type, neither alkylators nor IMiDs had a significantly reduced mortality. There was evidence of significant heterogeneity in the IMiDs group (I^2^ = 52%).

### 4 Progression

**Figure 3:**
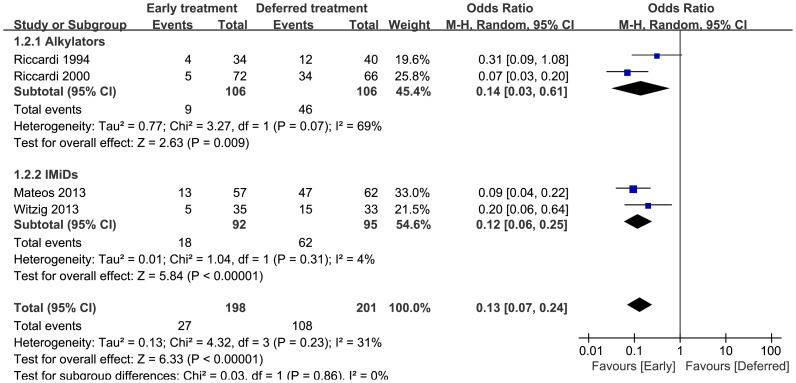
Progression rate odds ratios of early treatment versus deferred treatment. The 95% CIs are shown. Squares represent the odds ratios and 95% CI for alkylators or IMiDs and finally for the overall estimate. CIs, confidence intervals; IMiDs, immuno-modulatory agents.

In the early and deferred treatment arms, 198 and 201 patients were evaluable for disease progression, respectively. Twenty-seven (27) patients progressed to MM in the early treatment arm compared to 108 in the deferred treatment arm. As shown in Figure 3, there was a markedly reduced risk of disease progression with early treatment (pooled OR = 0.13, 95%CI = 0.07 to 0.24). No statistically significant heterogeneity was observed (p = 0.23, I^2^ = 31%). When divided by the treatment type, early treatment with either alkylators or IMiDs significantly delayed disease progression. There was evidence of significant heterogeneity in the alkylators group (I^2^ = 69%).

### 5 Response Rate

**Figure 4:**
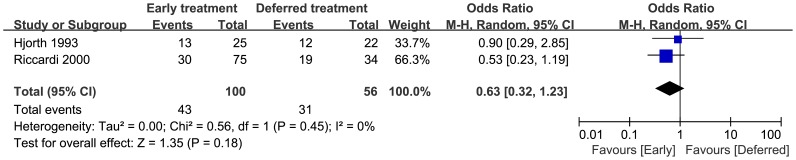
Response rate odds ratios of early treatment versus deferred treatment. Squares on the odds ratios plot are proportional to the weight of each study, which is based on the M-H method. Odds ratios are presents with 95% CIs. CIs, confidence intervals.

In the early and deferred treatment arms, 100 and 56 patients were evaluable for response rate, respectively. There were 43 responses in the early treatment arm and 31 responses in the deferred treatment arm. As shown in Figure 4, no heterogeneity was observed (p = 0.45, I^2^ = 0%). There was no significant difference in response rate between the two arms (pooled OR = 0.63, 95%CI = 0.32 to 1.23).

### 6 Adverse events

#### 6.1 Acute leukemia

**Figure 5:**
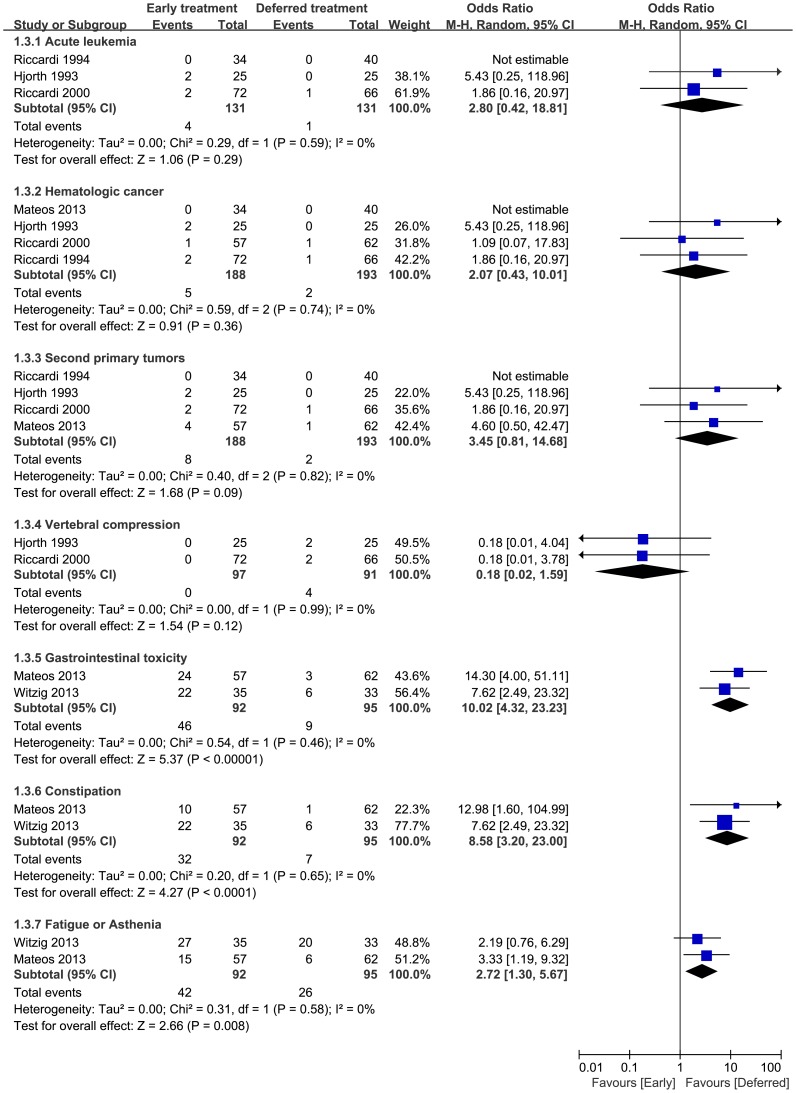
Adverse events rate odds ratios of early treatment versus deferred treatment. Squares on the odds ratios plot are proportional to the weight of each study, which is based on the M-H method. Odds ratios are presents with 95% CIs. CIs, confidence intervals.

Acute leukemia was reported in three trials. 4 of 131 patients in the early treatment arm and 1 of 131 patients in the deferred arm developed acute leukemia. No heterogeneity was observed (p = 0.59, I^2^ = 0%). The incidence of acute leukemia in the early treatment arm did not differ significantly from that in the deferred treatment arm (pooled OR = 2.80, 95%CI = 0.42 to 18.81) (Figure 5).

#### 6.2 Hematologic cancer

Hematologic cancer was reported in four studies. 5 of 188 patients in the early treatment arm and 2 of 193 patients in the deferred arm developed hematologic cancer. No heterogeneity was observed (p = 0.74, I^2^ = 0%). The incidence of hematologic cancer in the early treatment arm did not significantly differ from that in the deferred treatment arm (pooled OR = 2.07, 95%CI = 0.43 to 10.01) (Figure. 5).

#### 6.3 Second primary tumors

Second primary tumors were reported in four studies. 8 of 188 patients in the early treatment arm and 2 of 193 patients in the deferred arm developed second primary tumors. No heterogeneity was observed (p = 0.82, I^2^ = 0%). The incidence of second primary tumors did not significantly differ between the early and deferred treatment arms (pooled OR = 3.45, 95%CI = 0.81 to 14.68) (Figure 5).

#### 6.4 Vertebral compression

Vertebral compression was reported in two trials. None of the 97 patients in the early treatment arm, but 4 of 91 patients in the deferred treatment arm suffered from vertebral compression. No heterogeneity was observed (p = 0.99, I^2^ = 0%). The incidence of vertebral compression in the early treatment arm did not significantly differ from that in the deferred treatment arm (pooled OR = 0.18, 95%CI = 0.02 to 1.59) (Figure 5).

#### 6.5 Gastrointestinal toxicities

Gastrointestinal toxicities, including both constipation and diarrhea, were reported in only two the trials. Both studies reported the incidence of constipation, but only one reported the incidence of diarrhea. Forty-six (46) of 92 patients in the early treatment arm and 9 of 95 patients in the deferred treatment arm suffered from gastrointestinal toxicities. No heterogeneity was observed (p = 0.46, I^2^ = 0%). Patients in the early treatment arm experienced a greater incidence of gastrointestinal toxicities (pooled OR = 10.02, 95%CI = 4.32 to 23.23) (Figure 5).

#### 6.6 Constipation

32 of 92 patients in the early treatment arm and 7 of 95 patients in the deferred treatment arm suffered from constipation when only constipation among gastrointestinal toxicities was evaluated. No heterogeneity was observed (p = 0.65, I^2^ = 0%). We found significant differences between the two arms, with more patients in early treatment arm experiencing greater incidence of constipation (pooled OR = 8.58, 95%CI = 3.20 to 23.00) (Figure 5), but all the cases were grade 1 or 2.

#### 6.7 Fatigue or asthenia

Fatigue or asthenia was reported in two trials. 42 of 92 patients in the early treatment arm and 26 of 95 patients in the deferred treatment arm experienced fatigue or asthenia**.** No heterogeneity was observed (p = 0.58, I^2^ = 0%). We found significant differences between the two arms, with a greater incidence of fatigue or asthenia in the early versus deferred treatment arms (pooled OR = 2.72, 95%CI = 1.30 to 5.67) (Figure 5). Fatigue or asthenia at grade 1 or 2 was reported in 37 of 92 patients in the early treatment arm and 23 of 95 patients in the deferred treatment arm. Higher grade fatigue or asthenia (3 or 4) was reported in 5 of 92 patients in the early treatment arm and 3 of 95 patients in the deferred treatment arm.

## Discussion

The meta-analysis by He et al. in 2003 [5] evaluated three RCTs that used melphalan and prednisone to treat patients with SMM. Researchers have since questioned whether their conclusion that early treatment did not decrease mortality was actually a false-negative result. This was based on the fact that they only had 262 patients in their analysis, which was only 70% of the requisite 350 patients or more that were needed to reliably detect 15% mortality difference based on the Pogue and Yusuf formula they used. In our analysis, 449 patients were included, providing us more statistical power to evaluate this difference. However, even with a greater number of patients, there was no survival benefit in treating SMM patients early. This held true when the data were combined or when we analyzed the trials by treatment group, alkylators and IMiDs, respectively. Notably, doing this puts us under the same criticism of the work by He et al. because of the reduced number of patients in each group. More large-scale trials with IMiDs and newly emerging therapies are needed to provide more evidence.

Mhaskar et al. [20] performed a meta-analysis of 10 RCTs comparing efficacy of early versus late first-line treatments for cancer. They conducted subgroup analysis based on cancer type, including MM. Their subgroup analyses for MM showed no difference in survival and response rate. But there was statistically significant difference in progression-free-survival. These results were consistent with ours. But they did not compare safety of early versus late first-line treatments for MM, which is one important part in our analysis.

Only the Mateos trial [21], among all trials included here, showed that mortality was significantly lower in the early treatment arm compared to that of the deferred treatment arm (7% versus 21%; OR = 0.28, 95%CI = 0.09 to 0.93). Interestingly, although all the patients included in all the trials we analyzed had SMM, the patients in the Mateos trial included a large number of high risk SMM. The addition of the high risk SMM patients could contribute to the different clinical outcomes. This is, however, confounded because there has not been a consistent definition for high risk SMM [22]–[24], and none of the other trials selected patients with high risk SMM, so we could not address this question. Moving forward, it will be important not only to evaluate early treatment for patients with high-risk SMM, but also to consistently define high-risk SMM.

Along with no reduction in mortality, we also found that early treatment with alkylators did not significantly improve response rates. Notably, these data were available for only two trials. Since the response rate of the deferred treatment arm in these trials with IMiDs were not reported, we were not sure whether early treatment with IMiDs could improve response rates, comparatively.

Despite there being no difference in mortality or response rates between the early and deferred treatment arms, our meta-analysis demonstrated that early treatment decreased the risk of disease progression. This also held true when we stratified the trials by treatment with either alkylators or IMiDs. The fact that overall mortality is not reduced but progression is delayed indicates that the early treatment group survives for a shorter amount of time than the deferred treatment group once the disease has progressed. This suggests that early treatment might actually predispose toward a more aggressive, more difficult to treat MM disease once it has progressed. The addition of molecular analyses at both diagnosis and progression may help to explain this phenomenon.

In addition to efficacy, safety is an equally important consideration for whether to subject patients to early treatment. Only vertebral compression and acute leukemia were analyzed in the meta-analysis of 2003. Apart from these adverse events, we also extracted data on gastrointestinal toxicity, hematological cancer, second primary tumor, and fatigue or asthenia. Other adverse events, such as neuropathy, anemia, rash and infection were not consistently reported, and were not considered in this analysis. Although there was a trend for increased incidence of acute leukemia, hematologic cancer, second primary tumors and vertebral compression, our meta-analysis revealed that there was no statistically significant difference between the early and deferred treatment arms. There were few cases that reported these adverse events and follow-up time was limited. Therefore, our findings should be further verified with more RCTs and longer follow-up times. Analyses of gastrointestinal toxicities showed that early treatment with IMiDs was associated with an increased incidence. When only constipation was evaluated, we found an increased incidence for the early IMiD treatment group, but all the cases were moderate. Data on gastrointestinal toxicities were not available for trials that used alkylators. Data were integrated regarding fatigue and asthenia because they were frequently reported together. Early treatment with IMiDs appeared to increase the incidence of fatigue or asthenia, but most cases were moderate. Such data were not available for studies that used alkylators. Our findings call attention to gastrointestinal toxicities and fatigue or asthenia as the predominant safety concerns associated with treating SMM patients with IMiDs.

There were a number of limitations of this meta-analysis. First, all individual studies used in our analyses had small sample sizes and did not have predetermined power analyses. Due to the small sample sizes, the data on vertebral compression, acute leukemia, hematological cancer and second primary tumor were not interpretable. Second, only two of the included studies reported an adequate method for randomized allocation and allocation concealment. Third, sample sizes were also too small for reliable subgroup analyses for IMiDs versus alkylators. Fourth, separate subgroup analysis was not possible on low- versus high-risk SMM. Finally, statistically significant heterogeneity was seen in subgroup of progression (I^2^ = 69%) and in subgroup of mortality (I^2^ = 52%). This might be derived largely from potential differences in the proportion of different SMM risk stratification, non-uniform reporting of clinical parameters, and variability in clinical factors.

Despite these limitations, our meta-analysis demonstrated that early treatment did indeed delay disease progression, but delaying progression did not translate into an improved response rate or decreasing mortality. However, further RCTs were needed to determine if early treatment of SMM with the newer, targeted agents and/or patients characteristics were more likely to improve response rate and overall survival. Early treatment also resulted in an increased risk of gastrointestinal toxicities and fatigue or asthenia.

## Supporting Information

Table S1 **Search criterion of Medline (via Pubmed, from inception to May 16, 2014).**
(DOC)

Table S2 **Search criterion of Cochrane Library (from inception to May 17, 2014).**
(DOC)

Table S3 **Characteristics of excluded full-text studies.**
(DOC)

Checklist S1 **PRISMA checklist.**
(DOC)
