# Supplementary material for: Early versus Deferred Treatment for Smoldering Multiple Myeloma: A Meta-Analysis of Randomized, Controlled Trials
Source: PLoS One. 2014 Oct 3;9(10):e109758. doi: 10.1371/journal.pone.0109758 (PMC4184905; doi:10.1371/journal.pone.0109758)
Supplement: Table S1 — Search criterion of Medline (via Pubmed, from inception to May 16, 2014). (DOC) [file pone.0109758.s001.doc]

**Table S1 Search criterion of Medline (via Pubmed, from inception to May 16, 2014)**

| **No.** | **Query Results** | **Results** |
| --- | --- | --- |
| #15 | Search ((((((((((myeloma[Title/Abstract]) OR myelom*[Title/Abstract]) OR multiple myeloma[Title/Abstract]) OR plasmacytoma[Title/Abstract]) OR plasmocytom*[Title/Abstract])) OR "Plasmacytoma"[Mesh])) AND ((((((smoldering[Title/Abstract]) OR asymptomatic[Title/Abstract]) OR stage I[Title/Abstract]) OR early stage[Title/Abstract])) OR "Time Factors"[Mesh]))) AND ("Randomized Controlled Trial" [Publication Type] OR "Randomized Controlled Trials as Topic"[Mesh]) | 123 |
| #14 | Search "Randomized Controlled Trial" [Publication Type] OR "Randomized Controlled Trials as Topic"[Mesh] | 450219 |
| #13 | Search (((((smoldering[Title/Abstract]) OR asymptomatic[Title/Abstract]) OR stage I[Title/Abstract]) OR early stage[Title/Abstract])) OR "Time Factors"[Mesh] | 1161772 |
| #12 | Search "Time Factors"[Mesh] | 984105 |
| #11 | Search early stage[Title/Abstract] | 56293 |
| #10 | Search stage I[Title/Abstract] | 28395 |
| #9 | Search asymptomatic[Title/Abstract] | 108382 |
| #8 | Search smoldering[Title/Abstract] | 843 |
| #7 | Search ((((((myeloma[Title/Abstract]) OR myelom*[Title/Abstract]) OR multiple myeloma[Title/Abstract]) OR plasmacytoma[Title/Abstract]) OR plasmocytom*[Title/Abstract])) OR "Plasmacytoma"[Mesh] | 53864 |
| #6 | Search "Plasmacytoma"[Mesh] | 7514 |
| #5 | Search plasmocytom*[Title/Abstract] | 1379 |
| #4 | Search plasmacytoma[Title/Abstract] | 4718 |
| #3 | Search multiple myeloma[Title/Abstract] | 24307 |
| #2 | Search myelom*[Title/Abstract] | 46710 |
| #1 | Search myeloma[Title/Abstract] | 37080 |
