# Supplementary material for: Early versus Deferred Treatment for Smoldering Multiple Myeloma: A Meta-Analysis of Randomized, Controlled Trials
Source: PLoS One. 2014 Oct 3;9(10):e109758. doi: 10.1371/journal.pone.0109758 (PMC4184905; doi:10.1371/journal.pone.0109758)
Supplement: Table S2 — Search criterion of Cochrane Library (from inception to May 17, 2014). (DOC) [file pone.0109758.s002.doc]

**Table S2 Search criterion of** **Cochrane Library (from inception to May 17, 2014)**

| **No.** | **Query Results** | **Results** |
| --- | --- | --- |
| #1 | “myeloma”:ti,ab,kw or “myeloma*”:ti,ab,kw or “multiple myeloma”:ti,ab,kw or “plasmacytoma”:ti,ab,kw or “plasmocytom*”:ti,ab,kw (Word variations have been searched) | 2163 |
| #2 | MeSH descriptor: {Plasmacytoma} explode all trees | 9 |
| #3 | #1 or #2 | 2163 |
| #4 | “smoldering”:ti,ab,kw or “asymptomatic”:ti,ab,kw or “stage I”:ti,ab,kw or “early stage”:ti,ab,kw (Word variations have been searched) | 9999 |
| #5 | MeSH descriptor: [Time Factors] explode all trees | 50422 |
| #6 | #4 or #5 | 59635 |
| #7 | #3 and #6 | 156 |
