# Supplementary material for: Early versus Deferred Treatment for Smoldering Multiple Myeloma: A Meta-Analysis of Randomized, Controlled Trials
Source: PLoS One. 2014 Oct 3;9(10):e109758. doi: 10.1371/journal.pone.0109758 (PMC4184905; doi:10.1371/journal.pone.0109758)
Supplement: Table S3 — Characteristics of excluded full-text studies. (DOC) [file pone.0109758.s003.doc]

**Table S3** Characteristics of excluded full-text studies

| **Study*** | **Reason for exclusion** |
| --- | --- |
| Golombick T, 2012 [1] | Too small (17 SMM), no available data of treatment outcomes that defined in this meta-analysis† |
| Lipton A, 2012 [2] | Different comparison |
| D’Arena G, 2011 [3] | The early treatment drug is diphosphonate pamidronate, without additional chemotherapy or new-target drug. |
| Musto P, 2008 [4] | The early treatment drug is diphosphonate zoledronic acid, without additional chemotherapy or new-target drug. |
| Body JJ, 2006 [5] | Not SMM, data on skeletal events. |
| Rajkumar SV, 2003 [6] | Not RCT |
| Musto P, 2003 [7] | The early treatment drug is diphosphonate pamidronate, without additional chemotherapy or new-target drug. |
| He Y, 2003 [8] | Meta-analysis |

† The main outcome of our meta-analysis was mortality, progression, response rate, and adverse events.

SMM, smoldering multiple myeloma.

**References:**

1. Golombick T, Diamond TH, Manoharan A, Ramakrishna R. (2012) Monoclonal gammopathy of undetermined significance, smoldering multiple myeloma, and curcumin: a randomized, double-blind placebo-controlled cross-over 4g study and an open-label 8g extension study. Am J Hematol 87:455-460.
2. Lipton A, Fizazi K, Stopeck AT, Henry DH, Brown JE, et al. (2012) Superiority of denosumab to zoledronic acid for prevention of skeletal-related events: a combined analysis of 3 pivotal, randomised, phase 3 trials. Eur J Cancer 48:3082-3092.
3. D'Arena G, Gobbi PG, Broglia C, Sacchi S, Quarta G, et al. (2011) Pamidronate versus observation in asymptomatic myeloma: final results with long-term follow-up of a randomized study. Leuk Lymphoma 52:771-775.
4. Musto P, Petrucci MT, Bringhen S, Guglielmelli T, Caravita T, et al. (2008) A multicenter, randomized clinical trial comparing zoledronic acid versus observation in patients with asymptomatic myeloma. Cancer 113:1588-1595.
5. Body JJ, Facon T, Coleman RE, Lipton A, Geurs F, et al. (2006) A study of the biological receptor activator of nuclear factor-kappaB ligand inhibitor, denosumab, in patients with multiple myeloma or bone metastases from breast cancer. Clin Cancer Res 12:1221-1228.
6. Rajkumar SV. (2003) Thalidomide in newly diagnosed multiple myeloma and overview of experience in smoldering/indolent disease. Semin Hematol 40:17-22.
7. Musto P, Falcone A, Sanpaolo G, Bodenizza C, Cascavilla N, et al. (2003) Pamidronate reduces skeletal events but does not improve progression-free survival in early-stage untreated myeloma: results of a randomized trial. Leuk Lymphoma 44:1545-1548.
8. He Y, Wheatley K, Clark O, Glasmacher A, Ross H, et al. (2003) Early versus deferred treatment for early stage multiple myeloma. Cochrane Database Syst Rev D4023.
